# Cannabidiol use and effectiveness: real-world evidence from a Canadian medical cannabis clinic

**DOI:** 10.1186/s42238-021-00078-w

**Published:** 2021-06-23

**Authors:** Lucile Rapin, Rihab Gamaoun, Cynthia El Hage, Maria Fernanda Arboleda, Erin Prosk

**Affiliations:** Research Department, Santé Cannabis, 4150 Ste-Catherine O. Bureau 225, Montréal, QC H3Z 2Y5 Canada

**Keywords:** Cannabidiol, Symptoms, Medical cannabis, Effectiveness, Pain, Anxiety, Depression

## Abstract

**Background:**

Cannabidiol (CBD) is a primary component in the cannabis plant; however, in recent years, interest in CBD treatments has outpaced scientific research and regulatory advancement resulting in a confusing landscape of misinformation and unsubstantiated health claims. Within the limited results from randomized controlled trials, and lack of trust in product quality and known clinical guidelines and dosages, real-world evidence (RWE) from countries with robust regulatory frameworks may fill a critical need for patients and healthcare professionals. Despite growing evidence and interest, no real-world data (RWD) studies have yet investigated patients’ reports of CBD impact on symptom control in the common expression of pain, anxiety, depression, and poor wellbeing. The objective of this study is to assess the impact of CBD-rich treatment on symptom burden, as measured with a specific symptom assessment scale (ESAS-r).

**Methods:**

This retrospective observational study examined pain, anxiety, depression symptoms, and wellbeing in 279 participants over 18 years old, prescribed with CBD-rich treatment at a network of clinics dedicated to medical cannabis in Quebec, Canada. Data were collected at baseline, 3 (FUP1), and 6 (FUP2) month after treatment initiation. Groups were formed based on symptom severity (mild vs moderate/severe) and based on changes to treatment plan at FUP1 (CBD vs THC:CBD). Two-way mixed ANOVAs were used to assess ESAS-r scores differences between groups and between visits.

**Results:**

All average ESAS-r scores decreased between baseline and FUP1 (all *p*s < 0.003). The addition of delta-9-tetrahydrocannabinol (THC) during the first follow-up had no effect on symptom changes. Patients with moderate/severe symptoms experienced important improvement at FUP1 (all *p*s < 0.001), whereas scores on pain, anxiety, and wellbeing of those with mild symptoms actually increased. Differences in ESAS-r scores between FUP1 and FUP2 were not statistically different.

**Conclusion:**

This retrospective observational study suggests CBD-rich treatment has a beneficial impact on pain, anxiety, and depression symptoms as well as overall wellbeing only for patients with moderate to severe symptoms; however, no observed effect on mild symptoms. The results of this study contribute to address the myths and misinformation about CBD treatment and demand further investigation.

## Background

*Cannabidiol* (CBD) is one of the primary cannabinoids found in significant but variable concentrations in cannabinoid-based medicines (CBM). While structurally similar to *Δ9-tetrahydrocannabinol* (THC), CBD does not cause intoxication or euphoria (Russo 2017) and has showed considerable tolerability in humans with a low abuse potential (Chesney et al. 2020). This favorable safety profile has led to the recent mitigation of legal and regulatory barriers surrounding purified CBD products in several countries and recent increased interest in CBD treatments. While recent rulings clarified that CBD is not a drug under the 1961 United Nations as Single Convention on Narcotic Drugs, regulatory status in the USA remains extremely confusing. When derived from cannabis, CBD is a schedule 1 drug but when derived from “industrial hemp” plants it may be lawful federally (Corroon and Kight 2018; Corroon et al. 2020). In Canada, CBD is controlled under the Cannabis Act as are all cannabinoids, cannabis, and cannabis-derived products (Canada Go 2021). This regulatory status imparts restrictions and access obstacles for researchers.

CBD is widely touted as a panacea for a wide range of health problems and has been marketed as a dietary and “wellness” product (Russo 2017; Khalsa et al. 2020; Eisenstein 2019). CBD’s potential effects as an add-on therapy have been studied for social anxiety disorders, schizophrenia, non-motor symptoms in Parkinson’s disease, and substance use disorders (Bergamaschi et al. 2011; Crippa et al. 2019; McGuire et al. 2018; Millar et al. 2019; Prud’homme et al. 2015; Thiele et al. 2019; Leehey et al. 2020). However, the evidence of its effectiveness for indications other than drug-resistant pediatric epilepsy conditions remains very limited (Larsen and Shahinas 2020; Franco et al. 2020) and safety considerations such as drug-drug interactions associated with unsupervised use remain (Chesney et al. 2020; Freeman et al. 2019). Randomized controlled trials (RCTs) are limited in their rigorous design, population sample, and duration of observation making generalization of results and long-term data scarce. Therefore, real-world evidence (RWE) provides valuable insights and supplemental information about the use, safety, and effectiveness of CBD-based treatments (Graham et al. 2020).

RWE from retrospective analyses and patient registries shows that CBMs are used for pain (chronic, neuropathic), mental health conditions, cancer-related symptoms (nausea, fatigue, weakness), HIV/AIDS, and neurological conditions (Bonn-Miller et al. 2014; Gulbransen et al. 2020; Lintzeris et al. 2020; Lucas and Walsh 2017; Sexton et al. 2016; Waissengrin et al. 2015). Symptom control is the primary reason for use of CBM, with most patients looking to address unalleviated symptoms, perceived symptom intensity, and burden on health-related quality of life independently of primary diagnosis (Sexton et al. 2016; Waissengrin et al. 2015; Baron et al. 2018; Purcell et al. 2019; Swift et al. 2005; Webb and Webb 2014). The Edmonton Symptom Assessment Scale-revised version (ESAS-r) is a validated scale to assess symptom burden developed for use in oncology and palliative care (Hui and Bruera 2017), it has relevance to medical cannabis care as patients are often treated for similar symptom management (Good et al. 2019; Pawasarat et al. 2020). Specifically, studies showed self-perceived improvement in ESAS-r emotional symptoms (anxiety and depression) scores following CBM treatment in oncology patients, while pain and wellbeing symptoms showed no improvement (Good et al. 2019; Pawasarat et al. 2020). Yet, RWE on CBD-rich products is scarce (Goodman et al. 2020; Shannon et al. 2019). In addition, although careful titration and treatment adjustment after initiation is critical to symptom improvement and adverse effects care, current literature has failed to address this issue.

In this study, we investigated treatment with CBD-rich products within a dedicated clinical setting in Quebec, Canada, and the effects on a very common clinical symptom expression of pain and comorbid anxiety and depression symptoms, as well as the effect on overall wellbeing. We also examined the relevant clinical effects that were observed when CBD-rich treatments were replaced by THC:CBD-balanced products at subsequent follow-up visits.

## Methods

### Study population

This study is a retrospective examination of patients who were prescribed CBD-rich products by physicians at a clinic dedicated to CBM treatments operating at four locations across Quebec, Canada. All data are collected as part of standard clinical procedures during the initial visit and during 3 (FUP1) and 6 (FUP2) month follow-up visits and extracted from electronic medical records (EMR) (Prosk et al., 2021). All data were anonymized following extraction from the EMR and no identifiers linking to original data were maintained. A waiver of consent was required and approved by Advarra Ethics Committee, who also approved the study protocol, and by the provincial privacy commission (*La commission d*’*accès à l*’*information du Quebec*).

Adult patients, at least 18 years of age, who were initially treated exclusively with CBD-rich products from 1 October 2017 to 31 May 2019 and for whom outcome scores and product information were recorded at FUP1 were included in this study. Patients were generally referred by primary-care physicians and specialists for an assessment on the suitability of medical cannabis to treat refractory symptoms. A complete medical history, including primary and secondary diagnoses, was collected at baseline visit. Medical cannabis treatment decisions are determined at the discretion of a clinic physician according to a standardized clinical procedure, including symptom identification, selection of product format, cannabinoid profile, and dosage based on existing evidence (MacCallum and Russo 2018; Cyr et al. 2018), but also to minimize risk of adverse effects. Patient and physician preference may also indicate initiation with products that have higher CBD and lower THC concentration in order to limit use of THC and its inherent potential adverse events. The follow-up visits serve to assess treatment compliance, safety, and effectiveness.

### CBD-rich products in Canada

CBD-rich products are administered in various methods and formats, but most commonly as oral plant-derived extracts or oils and as inhaled dried flowers. In the Canadian medical cannabis program, CBD-rich cannabis oils contain approximately 0.5–1 mg of THC/mL and 20–25 mg of CBD/mL depending on the product manufacturer. Table 1 provides cannabinoid content and THC:CBD ratio for the three most common oil products (over 85% of patients) authorized at the clinic. Furthermore, product details in this study sample are described in Table 3. The clinic procedure dictates that all products with a ratio of CBD (mg) to THC (mg) higher than 10 are considered CBD-rich products.

**Table 1:** THC and CBD contents and associated THC:CBD ratio for the three most common oil products authorized at the clinic

	CBD-rich products at baseline			THC:CBD-balanced products at FUP1			THC-rich products at FUP1		
**Authorized dose range (in ml/intake)**	0.1–2			0.05–3			0.2–1.5		
**Oil (mg/ml)**	THC	CBD	Ratio THC:CBD	THC	CBD	Ratio THC:CBD	THC	CBD	Ratio THC:CBD
**Product 1**	1.2	24	1:25	9.5	12	10:13	27.5	< 1	30:1
**Product 2**	1.3	30	1:30	15	15	15:15	18.5	0.7	20:1
**Product 3**	< 1	20	1:20	10	13.5	10:13	26.3	< 1	30:1

The data is categorized by product category: CBD-rich products, THC:CBD-balanced products, and THC-rich products

*CBD* cannabidiol, *THC* Δ9-tetrahydrocannabinol, *SD* standard deviation

Treatment adjustments occur at follow-up visits as a result of lack of effectiveness, presentation of adverse effects, or social or economic barriers. Adjustments may include a change of the recommended CBD-rich product, method of administration, dosage, or a change in product formulation such as the introduction of THC:CBD-balanced or THC-rich products. We investigated the change from CBD-rich to THC:CBD products during FUP1 by forming two groups based on their product adjustment at FUP1 (CBD-rich vs THC:CBD). Products at FUP1 reflect those recommended at the visit. Therefore, the adjusted treatment affects only the evaluation at FUP2.

### Outcomes

Patients age, sex, and diagnosis were recorded at baseline. Patients completed the ESAS-r (Edmonton Symptom Assessment System-revised version) at each visit. The ESAS-r is a self-administered scale, rating the severity of symptoms from 0 (absence of symptom) to 10 (worst possible severity) at the time of assessment (Hui and Bruera 2017). Symptoms evaluated include six physical- (pain, tiredness, nausea, drowsiness, lack of appetite, and shortness of breath), two emotional- (depression, anxiety), and one overall wellbeing-related symptoms. ESAS scores can be categorized as mild (score 0 to 3) moderate (score 4 to 6) or high (score 7 and above) (Butt et al. 2008) and the threshold for clinically significant improvement is a decrease of 1 point (Hui et al. 2015). Since pain and mental health issues represent the most common symptoms for patients and physicians seeking medical cannabis treatments, we investigated effects on pain, depression, and anxiety symptoms as well as overall wellbeing. For each symptom, two groups of patients were formed: moderate-severe severity group in which a baseline score of 4 or more was recorded and a mild severity group with baseline score of 0 to 3.

### Analyses

Mean scores and standard deviation (SD), as well as percentage, where appropriate are presented for each variable. All analyses were performed on each ESAS-r symptom separately through the data analytics software R v4.0.2. An initial analysis compared the overall ESAS-r scores between each visit no matter the severity of the group, and looked at the role of product group (CBD/THC:CBD vs CBD/CBD group) (between-factor). Tukey HSD post hoc test was used to confirm where the differences occurred between groups.

To determine whether CBD-based treatments have different effectiveness based on the severity of patient symptoms, two-way mixed ANOVAs with severity group as between-factor and visit as a within-factor were conducted to assess the change in ESAS-r scores between visits. Paired t-tests were subsequently performed to assess the difference in mean scores within each severity group between baseline and FUP1. Significant *p* value was set at 0.05 and all analyses were two-tailed. Partial eta-squared (η^2^_p_) are reported to indicate magnitude of differences between groups.

## Results

### General

A total of 1095 patients were seen at the four clinic sites during the study period. Out of those, 715 were eligible for the study (at least 18 years old and initially treated exclusively with CBD-rich products). A total of 279 patients with ESAS-r scores and product information at FUP1 were analyzed (190 (68%) female, mean age = 61.1, SD = 16.6). The analyzed sample did not differ from the study-eligible group in terms of age, sex, or THC and CBD initial doses (all *p*s > 0.4). Table 2 outlines patient sample size and demographic information for each symptom and treatment group. Two hundred and ten (75%) patients were prescribed CBD-rich products to treat chronic pain, 19 (7%) for cancer-related symptoms, 21 (7.5%) to treat neurological disorders (Parkinson’s disease, multiple sclerosis, and drug-resistant epilepsy among others), 8 patients for inflammatory disease (arthritis), 10 for gastrointestinal disorders (Chron’s disease, inflammatory bowel syndrome, ulcerative colitis), 2 for anxiety, 1 for depression, 2 for headaches, and 6 unclassified. The chronic pain category included all medical indications for which pain was the main symptom such as but not limited to fibromyalgia, spinal stenosis, and chronic low back pain. Overall, 116 (41.6%) patients adjusted their prescription by adding THC at FUP1 (either to a THC:CBD-balanced combination or a THC-rich treatment). Two hundred and three (73%) patients had moderate/severe ESAS-r scores on at least 2 of the examined symptoms, 57 (20%) on three, and 75 (27%) on all four symptoms. Twenty-nine (10%) patients report no moderate/severe symptoms; these people may use CBD for other ESAS-r symptoms not examined here (shortness of breath, tiredness, nausea, drowsiness, appetite). There was no statistical difference on either age, sex, or THC and CBD initial doses between the patients who completed one FUP versus those who completed two FUP (all *p*s > 0.1).

**Table 2:** Demographic characteristics of 279 medical cannabis patients, by symptom group

	Sample size (percentage)	Number of female patients (percentage)	Mean age (SD)
Overall sample	279	190 (68)	61.1 (16.6)
Moderate or severe pain symptom group	205 (73.5)	150 (73)	61.8 (15.9)
Moderate or severe anxiety symptom group	138 (48.5)	97 (70)	61.43 (16.3)
Moderate or severe depression symptom group	115 (41.2)	81 (70)	60.5 (15)
Moderate or severe wellbeing group	202 (72.4)	141 (70)	60.8 (16.1)
CBD/THC:CBD group	116 (41.6)	75 (65)	60.38 (14.4)

The symptom groups are mild and moderate or severe. The table presents the moderate or severe demographic characteristics. The CBD/THC:CBD group is composed of patients who added THC to their CBD-rich prescription during FUP1

*CBD* cannabidiol, *THC* Δ9-tetrahydrocannabinol, *SD* standard deviation

### CBD-rich products characteristics

The baseline average daily doses for CBD and THC are presented in Table 3. The maximum initial CBD dose recorded (156 mg) was prescribed for the treatment of pain of one patient. The maximum THC dose recorded at FUP1 (90 mg) was prescribed for two patients for the treatment of pain.

**Table 3:** Details of the THC and CBD component of the CBD-rich, the THC:CBD 1:1, and the THC-rich formulations

	CBD-rich products at baseline (***n*** = 279)		THC:CBD-balanced products at FUP1 (***n*** = 104)		THC-rich products at FUP1 (***n*** = 12)	
	THC	CBD	THC	CBD	THC	CBD
Oil products (in mg/ml)	0.1–2.0	2.0–52.0	0.6–30	2.5–39	1.25–45	0–18
Dried flower (in % w/w)	0.7	17.0	3.7–9	7.7–13.4	13–27	0–0.5
Average daily dose (mg)	0.5	11.47	19.65	26.32	54.28	10.80
Standard deviation (mg)	0.43	10.21	5.80	9.12	29.65	7.64
Maximum daily dose (mg)	6	156	60	78	90	54

Data comes from our sample of 279 patients

*CBD* cannabidiol, *THC* Δ9-tetrahydrocannabinol

### Outcome of CBD treatment

**Fig. 1:**
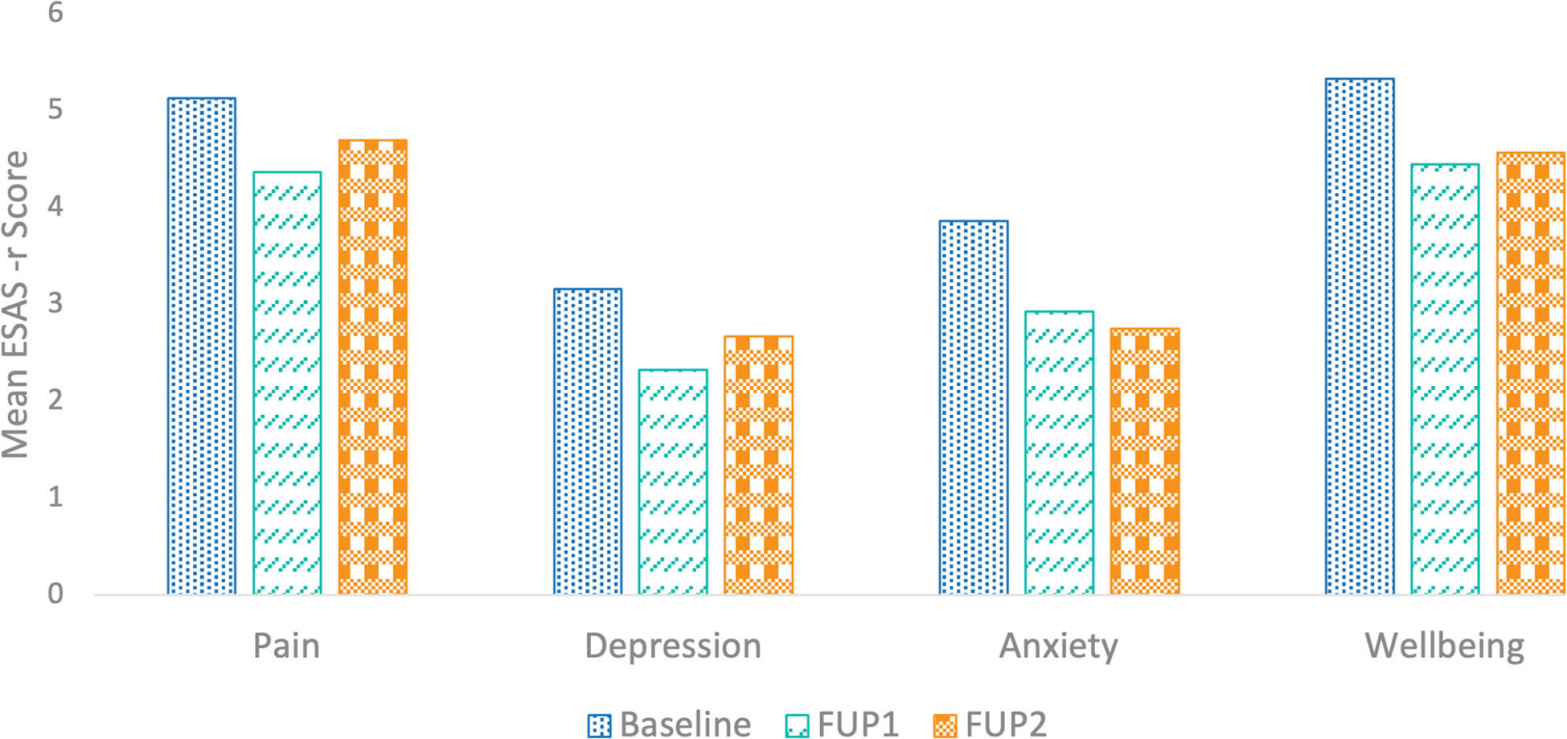
CBD-rich treatment effectiveness on pain, anxiety, depression symptoms, and on overall wellbeing in 279 patients. FUP1, follow-up visit at 3 month; FUP2, follow-up visit at 6 month. Mixed ANOVAs revealed a significant effect of visit on symptom reduction between baseline and FUP1 but not between FUP1 and FUP2

Mean ESAS-r scores of pain, anxiety, depression symptoms, and overall wellbeing at baseline, FUP1, and FUP2 are described in Table 4 and Fig. 1.

**Table 4:** Mean and standard deviation (SD) scores of ESAS-r scales for each severity group (mild or moderate/severe) and for each product group (CBD/CBD or CBD/THC:CBD)

Mean (SD)	Pain	Anxiety	Depression	Wellbeing
**Baseline (sample size)**	277	270	272	268
Overall sample	5.14 (2.57)	3.86 (3.19)	3.16 (3.08)	5.34 (2.61)
Mild severity group	1.69 (1.1)	0.99 (1.15)	0.87 (1.18)	1.86 (1.18)
Moderate or severe severity group	6.34 (1.7)	6.61 (1.78)	6.3 (1.86)	6.47 (1.83)
CBD/CBD group	5.03 (2.66)	3.80 (3.21)	2.99 (3.04)	5.28 (2.72)
CBD/THC:CBD group	5.28 (2.45)	3.95 (3.17)	3.40 (3.13)	5.42 (2.46)
**FUP1 (sample size)**	262	261	261	254
Overall Sample	4.37 (2.73)	2.93 (2.95)	2.33 (2.79)	4.45 (2.6)
Mild severity group	2.3 (2.4)	1.62 (2.08)	1.12 (1.78)	3.73 (2.75)
Moderate or severe severity group	5.04 (2.49)	4.15 (3.09)	3.77 (3.07)	4.72 (2.5)
CBD/CBD group	4.09 (2.67)	2.74 (2.87)	2.23 (2.71)	4.43 (2.6)
CBD/THC:CBD group	4.75 (2.78)	3.2 (3.05)	2.47 (2.9)	4.49 (2.63)
**FUP2 (sample size)**	101	99	102	97
Overall Sample	4.7 (2.7)	2.85 (3.01)	2.67 (3.02)	4.57 (2.47)
Mild severity group	2.18 (2.43)	1.32 (1.89)	1.52 (2.31)	3.82 (2.81)
Moderate or severe severity group	5.2 (2.47)	3.96 (3.19)	3.74 (3.26)	4.93 (2.23)
CBD/CBD group	4.55 (2.6)	2.44 (2.68)	2.44 (2.82)	4.76 (2.22)
CBD/THC:CBD group	4.88 (2.81)	3.08 (3.07)	2.94 (3.24)	4.36 (2.73)

The CBD/THC:CBD group is composed of patients who added THC to their CBD-rich prescription during FUP1. ESAS-r scores varied between 0 and 10 for all assessed symptoms and all visits except for the anxiety scale at FUP2 for which the maximum score was 9

*CBD* cannabidiol, *FUP1* follow-up visit at 3 month, *FUP2* follow-up visit at 6 month, *THC* Δ9-tetrahydrocannabinol

All average ESAS-r scores decreased between baseline and FUP1 and FUP2. This was further demonstrated by ANOVAs which revealed a significant effect of visit on mean ESAS-r scores for each symptom assessed (pain: F(2,634) = 4.9, *p* < 0.008; anxiety: F(2,624) = 8.36, *p* < 0.001, depression: F(2,629) = 5.36, *p* < 0.004; wellbeing: F(2,613) = 8.31, *p* < 0.001; all η^2^_p_ between 0.008 and 0.02). In all assessed symptoms, no significant main effect of adding THC at FUP1, nor visit-by-product interaction, were observed (all *p*s > 0.2). Post hoc tests revealed ESAS-r mean scores significantly decreased between baseline and FUP1 (all ps < 0.003) for all symptoms, between baseline and FUP2 for anxiety and wellbeing (both ps < 0.03), but not between FUP1 and FUP2 for any symptoms (all ps > 0.5). This suggests statistical improvement recorded at FUP1 is still present at FUP2 in all symptoms independently from treatment adjustment at FUP1.

### CBD treatment impact according to symptom severity

From Table 2, moderate or severe scores at baseline were most common for pain (205 patients, 73.5%) and poor wellbeing (202 patients, 72.4%).

**Fig. 2:**
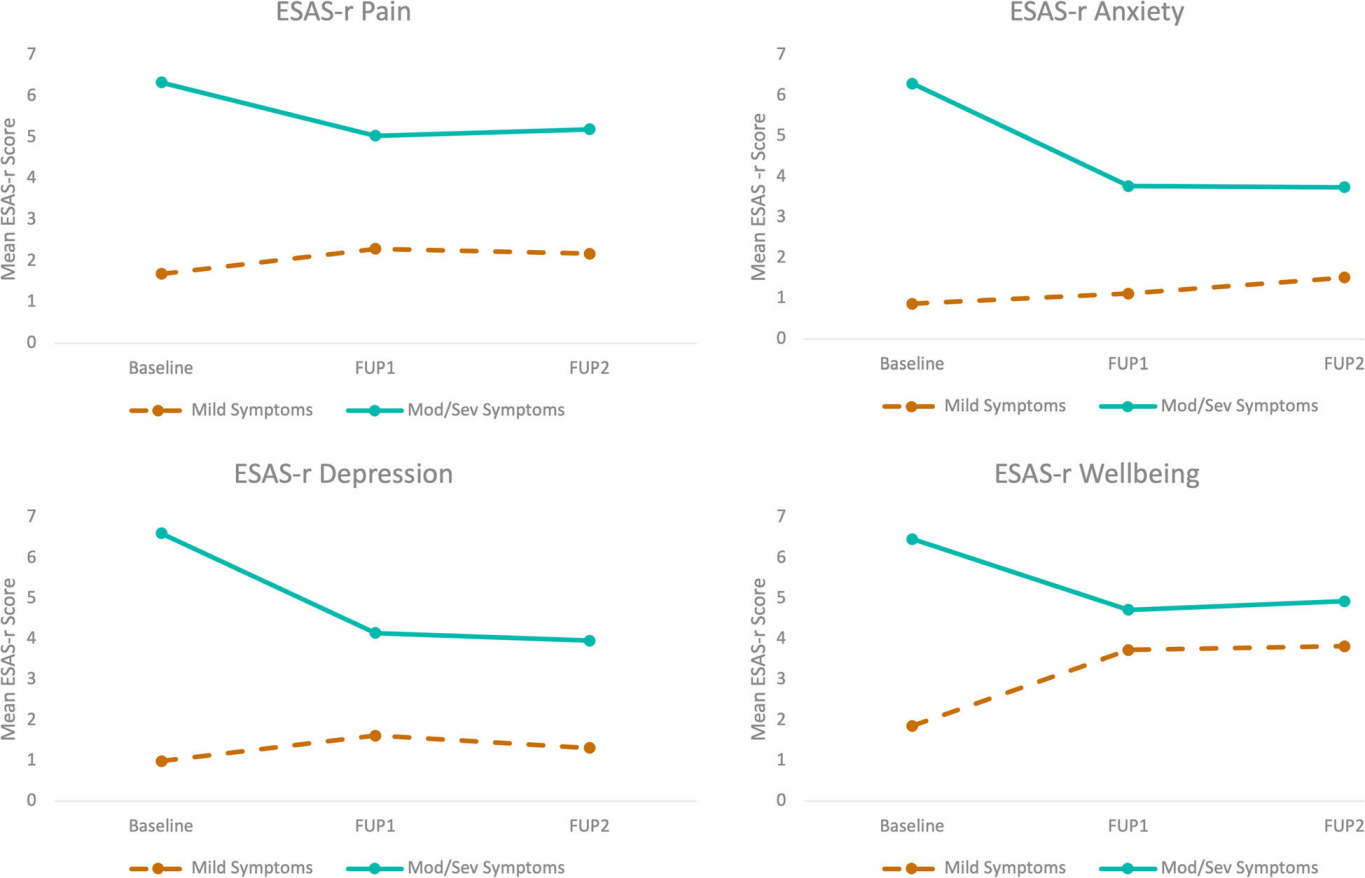
CBD-rich treatment effect according to symptom severity: mild or moderate/severe in 279 patients. FUP1, follow-up visit at 3 month; FUP2, follow-up visit at 6 month. **a** Mean ESAS-r scores for the pain symptom, **b** mean ESAS-r scores for the anxiety symptom, **c** mean ESAS-r scores for the depression symptom, and **d** mean ESAS-r scores for overall wellbeing. According to mixed ANOVAs, patients with moderate/severe symptoms reported symptom reduction whereas patients with mild symptoms reported symptom deterioration from baseline to FUP1. No effect was statistically significant between FUP1 and FUP2

Clinical effect (difference of 1.3 to 2.5 points) observed in all symptoms for patients with moderate/severe symptoms between baseline and FUP1; however, there was no clinical effect for patients with mild symptoms (from − 0.3 to − 1.8) (Fig. 2). No clinical effect was observed in any symptoms between FUP1 and FUP2 for patients with moderate/severe symptoms (− 0.4 to 0.5) as well as for patients with mild symptoms (from − 0.7 to 0.4).

### Pain

The ANOVA revealed that all main and interaction effects were significant at the 0.001 level with effect sizes large for severity (η^2^_p_ = 0.29), medium for visit (η^2^_p_ = 0.06), and small for the interaction (η^2^_p_ = 0.03). Post hoc tests revealed a significant score difference between baseline and FUP1 and FUP2 (both ps < 0.05) but not between FUP1 and FUP2 (*p* = 0.98). Patients with moderate/severe symptoms on pain experienced important improvement at FUP1 (t(194) = 7.61, *p* < 0.001) whereas ESAS-r scores for patients with mild symptoms actually increased (t(64) = − 2.03, *p* < 0.05) (Fig. 2a).

### Anxiety

There were significant effects of visit, severity group, and visit by group interaction (all *p*s < 0.001; η^2^_p_ = 0.006, η^2^_p_ = 0.4, η^2^_p_ = 0.1, respectively). Post hoc tests revealed a significant score difference between baseline and FUP1 and FUP2 (both *p*s < 0.001) but not between FUP1 and FUP2 (*p* = 0.38). Although there was a large improvement for patients with moderate to severe anxiety symptoms (t(131) = 9.36, *p* < 0.001), the anxiety scores of patients with mild symptoms increased (t(119) = − 3.19, *p* < 0.01) from baseline to FUP1 (Fig. 2b).

### Depression

The ANOVA showed main effects of visit, severity group (both *ps* < 0.001 with η^2^_p_ = 0.04 and η^2^_p_ = 0.4, respectively) and a significant group-by-visit interaction (F(2,620) = 34.47, *p* < 0.001; η^2^_p_ = 0.1). Post hoc tests revealed a significant score difference between baseline and FUP1 and FUP2 (both *p*s < 0.01) but not between FUP1 and FUP2 (*p* = 0.85). Specifically, the scores of moderate/severe group decreased notably (t(110) = 9.56, *p* < 0.001) between baseline and FUP1 but the scores of the group with mild depression symptoms did not (*p* = 0.07) (Fig. 2c).

### Wellbeing

The ANOVA showed main effects of visit, severity group (both *ps* < 0.001 with η^2^_p_ =0.04 and η^2^_p_ =0.3 respectively) and a significant group-by-visit interaction (F(2,597) = 36.53, *p* < 0.001; η^2^_p_ = 0.11). Post hoc tests revealed a significant main score difference between baseline and FUP1 and FUP2 (both *p*s < 0.01) but not between FUP1 and FUP2 (*p* = 0.89). Precisely, the scores of the group reporting good wellbeing increased (t(182) = 8.8, *p* < 0.001) whereas scores of patients with worst wellbeing notably decreased (t(59) = − 5.08, *p* < 0.001) between FUP1 and FUP2 (Fig. 2d).

## Discussion

This retrospective study explored the use of CBD-rich products in a medical cannabis clinical setting in Canada and associated effectiveness on a common symptom cluster presentation of pain, anxiety, depression, and poor sense of wellbeing, as measured by ESAS-r.

Patients treated with CBD-rich products were mainly women in their sixties, seeking predominantly chronic pain management.

Our findings show that overall effectiveness of CBD treatment is primarily by patients with moderate to severe symptoms. A deficiency in the endocannabinoid system (ECS) may provide a possible explanation for this result (Russo 2016). The ECS could be more deficient in patients with moderate/severe symptoms compared to mild symptoms leading to increased improvement in the first group. The absence of significant improvement for patients with mild symptoms at baseline may be explained by a smaller margin for symptom improvement. In such patients, CBD treatments may have been targeted to other clinical symptoms not assessed in the current study. There is a probable placebo effect; however, there were no differences in initial CBD doses between the severity groups. Furthermore, associated placebo effect would likely be decreased by FUP3M, also considering the significant treatment cost. The distinct beneficial impact of CBD treatment observed for patients with moderate-severe symptoms could elucidate discrepancies found in the literature.

RCTs on CBM and pain symptoms provide inconclusive results; however, several report that treatments of THC and CBD have some benefit for pain management (Häuser et al. 2018; Russo 2008; Prosk et al. 2020). Our results are largely novel as research on the effect of CBD on pain control is very limited (Boyaji et al. 2020). The reduction in reported anxiety may also contribute to the improvement in pain perception.

Discrepancies still exist regarding the anxiolytic effect of CBD. Some RCTs indicate an anxiolytic effect of CBD upon experimentally induced scenarios (Bergamaschi et al. 2011; Zuardi et al. 2017; Bhattacharyya et al. 2010; Skelley et al. 2020); however, these findings are difficult to replicate (Larsen and Shahinas 2020; Hundal et al. 2018; Crippa et al. 2012). This reinforces our findings that CBD may have a differential effect depending on anxiety severity. Regarding the effects of CBD on depression symptoms, further research is required to draw conclusions (Khalsa et al. 2020; Schier et al. 2014; Turna et al. 2017).

The addition of THC to CBD during FUP1 did not produce any effect on ESAS-r scores at FUP2 in this analysis; however, the magnitude of the difference between groups is small. The examination of treatment regimen has been seldom addressed in the literature and further development is required to inform guidelines for prescription and refinement of clinical practice.

Furthermore, a significant discrepancy is observed between the recorded dosages of oral CBD in RCTs and dosages in real-world settings. The average daily CBD dosage authorized at our clinic (11.5 mg) is closer to other observational studies (Gulbransen et al. 2020) compared to what is seen in RCTs (up to 1000 mg for a single dose) (Larsen and Shahinas 2020). The presence of THC and other cannabinoids in CBD-rich products may affect the outcomes in this study. The majority of RCTs investigated single-dose administration of CBD making it difficult to compare observed treatment outcomes with chronic dosing clinical settings. Importantly, medical cannabis products are generally not covered by most insurers and patients rely on out-of-pocket payments. The cost of CBD remains very high globally, approximately $CAD 5–20 per 100 mg (Canada Go 2021; Eisenstein 2019; Canada 2020). Availability of reliable cannabinoid testing in certain international jurisdictions is also limited. The gap between effective doses demonstrated in RCTs and the actual affordable doses demonstrated by RWE mandate the need for a precise pricing and marketing strategy at the initiation of any drug development process.

### Limitations

Limitations are common in real-world data (RWD), especially in retrospective studies. In this study, with no control group, no causality effect can be drawn between CBD-rich treatment and symptom improvement. Most patients treated with CBM present with multiple severe symptoms and the analyses presented here are limited to identify the treatment outcomes for such patients. Further studies can investigate the use of CBD to treat several symptoms simultaneously.

The self-reported subjective assessment used may be biased by the patient’s positive expectation of treatment, which could lead to a possible placebo effect. This perceived effectiveness bias may also be increased by social and economic barriers. The current context of medical cannabis access, including social stigma, high cost, and lack of universal insurance coverage can increase the patient selection bias. Self-selection bias is increased by the significant patient interest in medical cannabis as these patients must be motivated to access the non-traditional medication system. This bias limits the generalizability of results but is common across international medical cannabis regimens and should not discount the observed results. The heterogeneity of the patient population with a variety of diagnoses and the diversity of medical cannabis preparations also affects the external validity of the study. However, clinical findings from within Canada’s controlled regulatory program do provide important models for international consideration. Future research is required in controlled clinical settings to examine these factors in order to provide a more complete account of CBD effectiveness.

Also, there was a large drop of sample size (53% loss) due to missing data. Additionally, there was an important loss to follow-up at the 6-month visit (FUP2) due to missed appointment and cost barriers, limiting the power of the findings. The total treatment cost has significant impact on treatment continuation. Improved patient retention and more robust, harmonized data collection methods will improve future observational studies and allow for long-term assessment. Collection of detailed, accurate product information is a challenge, especially with inhaled products (Corroon et al. 2020). There are opportunities for administration devices and other technology advancements to improve this limitation. Lastly, this study did not include safety data assessment, future studies should investigate safety considerations of CBD (Chesney et al. 2020). Collection of high-quality RWD will require improvements in patient retention, data monitoring, and more robust data collection methods within a controlled clinical setting.

## Conclusion

This study on CBD-rich products demonstrates the potential of RWE for the advancement of medical cannabis research and practice guidelines, especially in a world where CBD use is exponentially increasing but scientific data are limited. It revealed that CBD-rich treatments have a beneficial impact on patients with self-reported moderate or severe symptoms of pain, anxiety, or depression and overall wellbeing but not in patients with mild symptoms. Further investigation is clearly required, but as of now the hyped, and often illegal, marketed claims of CBD as a wellness product are unsubstantiated. Our findings have important and novel implications to clinical practice, especially the examination of treatment plan adjustment during the first follow-up after initiation with CBD treatments. Improvements in access regimes, oversight, and clarification from regulatory agencies are also needed to improve the validity of RWE and assessment of the use of CBD-rich products.

## Data Availability

The datasets used and/or analyzed during the current study are available from the corresponding author on reasonable request.

## Abbreviations

CBD: Cannabidiol
CBM: Cannabinoid-based medicines
EMR: Electronic medical record
ESAS-r: Edmonton Symptom Assessment System-revised version
FUP: Follow-up visit
RWD: Real-world data
RWE: Real-world evidence
SD: Standard deviation
THC: Δ9-Tetrahydrocannabinol
